# Hyperferritinemia, Low Circulating Iron and Elevated Hepcidin May Negatively Impact Outcome in COVID-19 Patients: A Pilot Study

**DOI:** 10.3390/antiox11071364

**Published:** 2022-07-14

**Authors:** Robert Szabo, Cristina Petrisor, Constantin Bodolea, Robert Simon, Ioana Maries, Sebastian Tranca, Teodora Mocan

**Affiliations:** 1 Physiology Department, “Iuliu Hatieganu” University of Medicine and Pharmacy, 400000 Cluj-Napoca, Romania; szabo.robert@elearn.umfcluj.ro (R.S.); teodora.mocan@umfcluj.ro (T.M.); 2 Anaesthesia II Department, “Iuliu Hatieganu” University of Medicine and Pharmacy, 400000 Cluj-Napoca, Romania; petrisor.cristina@umfcluj.ro (C.P.); robert.simon@umfcluj.ro (R.S.); ioana.silvia.maries@gmail.com (I.M.); tranca.sebastian@umfcluj.ro (S.T.); 3 Clinical County Emergency Hospital, 400000 Cluj-Napoca, Romania; 4 Municipal Clinical Hospital, 400139 Cluj-Napoca, Romania; 5 Nanomedicine Department, Regional Institute of Gastroenterology and Hepatology, 400000 Cluj-Napoca, Romania

**Keywords:** COVID-19, iron overload, inflammation, hepcidin, interleukin 6, ferritin, oxidative stress, ferroptosis, anemia

## Abstract

Inflammation in COVID-19 produces intracellular iron overload with low circulating iron available for metabolic processes. The accumulated intracellular iron generates reactive species of oxygen and results in ferroptosis, a non-programmed cell death. Since no organ is spared, iron dysmetabolism increases the mortality and morbidity. Hepcidin and the mediator interleukin 6 are believed to play a role in the process. Our aim is to evaluate the predictive values of serologic iron and inflammatory parameters in COVID-19 critically ill patients. Hence, 24 patients were included. Hepcidin and interleukin 6, along with routine blood parameters, were determined and outcomes, such as death, multiple organ damage (MOD), anemia, and need for transfusions, were assessed. The results of this pilot study indicate that iron metabolism parameters individually, as well as models consisting of multiple laboratory and clinical variables, may predict the outcomes. Further larger studies are needed to validate the results of this pilot stud. However, this paper identifies a new direction for research.

## 1. Introduction

Iron is an essential ion indispensable for biological processes in living organisms. In humans, erythropoiesis is the biggest consumer of this element but is not limited to it; for example, iron is involved in nucleic acid synthesis. The latter is essential for bacteria and viruses alike [1]. As a result, iron is tightly controlled by complex mechanisms. Homeostasis is achieved by the interaction between hepcidin, an iron regulating protein synthesized by the liver, and the iron exporter ferroportin. The hepcidin-ferroportin complex is internalized within the cell, rendering the iron channel inactive. This mechanism is efficient because ferroportin is the sole exporter of intracellular iron [2].

The hepcidin feedback loop is not only controlled by iron levels alone. Hepcidin synthesis is also stimulated by inflammation through interleukin 6 (IL 6) and signal transducer and activator of transcription 3 (STAT3) pathways [3]. This mechanism is frequently seen in critically ill patients due to the presence of a systemic inflammatory response syndrome [4]. As a result of inflammation, a state of cellular iron overload with low circulating iron develops.

The outbreak of the COVID-19 pandemic brought to light the devastating effects of iron overload on the human body [5]. The ferrous (2+) form of iron can take part in oxidative stress reactions which produce free radicals and subsequent cell death. This is termed ferroptosis [6]. Since iron can accumulate virtually in any cell, no organ is spared from damage caused by hyperferritinemia and ferroptosis [5].

Apart from organ dysfunction, anemia is a big issue in critically ill patients due to the high morbidity and mortality associated with low hemoglobin (Hb) as well as with transfusions [7]. The mechanisms of anemia are manyfold, and some are not yet fully understood. Inflammation, via hepcidin induction, kidnaps iron needed for erythropoiesis. Of note, hepcidin may also be released from hepatocytes and macrophages through non-conventional pathways in the critically ill [8].

Studying the iron status as well as the mediators could shine light on the metabolic processes present in COVID-19 infected patients. It is important to identify patients at risk for complications or death. With a better understanding of iron regulatory pathways, we might identify prognostic, diagnostic and therapeutic approaches.

In this prospective observational pilot study, our objective is to simultaneously assess the probability of standard iron studies and of the iron regulator hepcidin to predict the occurrence of death, multiple organ dysfunction (MOD), anemia and need for transfusions. We also aim to assess models based on multiple laboratory and clinical variables to set a direction for future, larger studies.

## 2. Materials and Methods

We used a prospective cross-sectional design. The study was approved by the ethics committees of “Iuliu Hatieganu” University of Medicine and Pharmacy Cluj-Napoca (no. 140/06.05.2019) and of the Clinical County Emergency Hospital Cluj-Napoca (no. 8961/05.04.2019). Severe or critically ill patients were admitted to the intensive care unit (ICU) of the Emergency Clinical County Hospital Cluj, Cluj-Napoca, Romania, from February 2020 to December 2020 and were eligible for analysis. Severe cases were peripheral oxygen saturation below 94% despite supplemental oxygen or inspired fraction of oxygen ratio (P/F) below 300, clinical signs of respiratory difficulty such as tachypnoea > 30 breaths per minute with dyspnea, >50% lung infiltrate. Critically ill patients were considered those who presented respiratory failure, hemodynamic instability requiring vasoactive or inotropic support or the presence of multiple organ dysfunction [9]. Mild and moderate cases were treated on wards and were not admitted to ICU. Comorbidity was quantified after admission using Acute Physiology and Chronic Health Evaluation II score and Charlson Comorbidity Index. All patients had a positive real time polymerase chain reaction (RT-PCR) test confirming infection with the COVID-19 virus.

Before laboratory analysis, consent was obtained from conscious patients or their next of kin, in cases where mental status was altered. Adult patients aged 18 years or above who required ICU treatment for more than 48 h were included.

Exclusion criteria were: refusal to participate, anemia on admission or history of blood transfusions during the previous 7 days, undergoing treatment with iron supplements or chelators, history of severe renal disease (glomerular filtration rate < 30 mL/min) or undergoing chronic dialysis, history of diseases associated with iron metabolism disturbances, pregnant or breastfeeding patients, history of systemic inflammatory diseases, hematologic malignancies or diseases including coagulopathies, history of current chemotherapy or diagnosed viral infection (other than COVID-19).

After inclusion, patients underwent treatment and bundles of care as per hospital protocol which were adapted to patients’ clinical status. Hospital protocol for COVID treatment included antiviral treatment with Remdesivir (if within 7 days of symptom onset and administered for 10 days) or Favipiravir (if Remdesivir was not appropriate or unavailable and administered for 14 days), steroid therapy with Dexamethasone (all patients received dexamethasone 6mg per day for a period of 10 days), immunosuppressor therapy with Tocilizumab (in absence of sepsis and IL 6 levels above 100 pg/mL) or Anakinra (in absence of sepsis, ferritin > 1000 ng/mL and IL 6 < 100 pg/mL), and anticoagulant therapy with Enoxaparin (all patients received prophylactic anticoagulation or curative when indicated by the presence of thrombosis or underlying disease). Patients were prospectively observed for a period of 28 days, until discharge from ICU or death, whichever occurred first. The primary endpoint was death with subsequent endpoints being MOD, anemia and transfusions.

Blood samples were collected at regular intervals on days 0, 3, 7, 10, 14, 17, 21, 24, and 28. Cases where blood sampling coincided with ongoing or was within 24 h of continuous renal replacement therapy, were excluded from the study. For hepcidin and IL 6 quantification, serum was obtained from whole blood collected in standard biochemistry vacutainer, which was let to set for 30 min followed by centrifugation at 5000× *g* for 5 min. Serum was divided in 5 aliquots of 100 μL samples and stored at −80 °C until the end of the inclusion period. Routine blood works were performed, same day, as per standard hospital protocol in our hospital’s accredited laboratory. Blood sample tubes with EDTA were used for full blood count and with citrate for coagulation screen and fibrinogen. For biochemistry and immunology, tubes without anticoagulants were used. After sampling the tubes were immediately transported to the laboratory and handled accordingly. All parameters except for hepcidin and IL 6 were considered standard. Patients were also followed up clinically and, depending on the patient’s development and the clinician’s judgment, further tests were performed such as: cultures, computer tomography (CT), angiography.

The secondary endpoint was the development of multiple organ dysfunction and anemia. MOD was considered when 2 or more organ dysfunctions were diagnosed. Organ dysfunction was defined: respiratory by P/F below 300 requiring invasive mechanical ventilation; cardiac dysfunction by serum troponin levels above 23.2 pg/mL; hematological by platelets count below 150 × 10^9^/L; neurological dysfunction by Glasgow coma scale < 15; renal dysfunction by creatinine levels above 1.2 mg/dL; hepatic by total bilirubin levels > 1.2 mg/dL [10]. Anemia was defined by Hb levels below 13 g/dL in males and 12 g/dL in females. Severe anemia requiring transfusions was defined as: (i) Hb < 6 g/dL; (ii) Hb 6–8 g/dL associated with either tachycardia, hypotension, ischemic changes on EKG, lactic acidosis, low central venous oxygen saturation or reduced compensatory reserve (ischemic heart disease, cardiac failure, cerebro-vascular disease); (iii) Hb 8–10 g/dL associated with reduced compensatory reserve [11]. Bacterial supra-infection was defined as the combined presence of clinical features (temperature > 38 °C or <36 °C, imaging describing consolidations, moderate polymorphonuclears on sputum gram staining) and microbiological criteria (pathogen identified at any sites). Likewise, a probable superinfection was considered when clinical features were present and improved under antimicrobial therapy [12].

Quantitative IL 6 and hepcidin levels were determined from the stored serum using human IL 6 Quantikine and human hepcidin standard ELISA kits. In short, both parameters were assessed using standard quantitative sandwich enzyme immunoassay technique.

For IL 6, a calibration curve was constructed by the reconstitution of standard with Calibrator Diluent RD5T, followed by the performance of seriate dilutions, according to protocol. The addition of the indicated assay diluent volume (100 μL) in each well, followed by insertion of the standard, control, or sample (100 μL) was carried out in the provided 96-well plate. After 2-h incubation and an intermediate washing step, the addition of Human IL 6 Conjugate was performed, followed by incubation (2 h, RT) and a washing/aspiration step. Consequently, incubation with 200 μL of Substrate Solution was carried out (20 min, RT, protected from light). The reaction was further stopped with 50 μL of stop solution, followed by OD determination within the next 30 min (540 nm).

For hepcidin, reconstitution of standard and of standard diluent was carried out and calibration curve was constructed according to protocol. Next, 50 μL of standard or 40 μL of sample + 10 μL anti-HEPC antibody was added, respectively. Subsequently, sample wells and standard wells were supplemented with 50 μL streptavidin-HRP and incubated (60 min, 37 °C). After proceeding to a washing step, substrate solution was added (50 μL of solution A, 50 μL of solution B), followed by incubation (10 min, 37 °C, dark room). The reaction was stopped (50 μL of stop solution) and OD was determined within the next 10 min (450 nm).

Iron level was determined using the TPTZ (2,4,6-Tri-(2-pyridyl)-5-triazine) chromogen method. In an acidic medium, transferrin-bound iron dissociates into free ferric ions and apo-transferrin. Hydrochloric acid and sodium ascorbate reduced the ferric ions to the ferrous state. Next, the ferrous ions were then reacted with TPTZ to form a blue coloured complex which was measured bichromatically at 600/800 nm. The increase in absorbance was considered directly proportional to the amount of iron present.

Ferritin determination was carried out using latex agglutination reactions occurring as a result of antibody-coated latex beads aggregating if antigen is present in sufficient quantity. Immune complexes formed in solution scatter light in proportion to their size, shape, and concentration. Under conditions of antibody excess, increasing amounts of antigen result in higher scatter. Turbidimeters measure the reduction of incident light due to reflection, absorption, or scatter. The measurement of the decrease in light intensity transmitted (increase in absorbance) through particles suspended in solution as a result of complexes formed during the antigen-antibody reaction, is the basis of this assay. The Ferritin reagent is a suspension of polystyrene latex particles, of uniform size, coated with polyclonal rabbit anti-ferritin antibody. The sample containing ferritin, when mixed with the Ferritin reagent, generates an agglutination mixture. This was measured spectrophotometrically on a Beckman Coulter Chemistry Analyser.

Immuno-turbidimetric test for the quantitative determination of C-reactive protein (CRP) in human serum and plasma was performed using a Beckman Coulter AU analyser. The sample was mixed with R1 buffer and R2 latex suspension, CRP reacted specifically with anti-human CRP antibodies coated on the latex particles to yield insoluble aggregates. The absorbance of these aggregates was proportional to the CRP concentration in the sample.

Data were analyzed for distribution followed by the appropriate statistical analysis to test for significant differences between groups. Numerical values were expressed as mean (± standard deviation) or median (interquartile range), according to normality of data, as assessed using Kolmogorov–Smirnov and Shapiro–Wilk tests. For the assessment of the predictive value of various continuous variables, receiver operating characteristic curves (ROC) were used, along with area under the curve determination (AUC). Specificity and sensibility were calculated for detected cut-off values. Between-group differences for non-continuous data were tested using the Chi-square (X^2^) or Fisher exact tests, where appropriate according to standard conditions. Differences in continuous numerical parameters were tested using the Student t test or Mann–Whitney U test, with test selection according to normality results. Finally, multiple logistic regression was adopted to predict the probability of the primary outcome to occur. Models were based on combinations of different laboratory and clinical independent variables. The statistics software package Prism-GraphPad 9 was used for all data analyses.

## 3. Results

### 3.1. Participants

**Figure 1:**
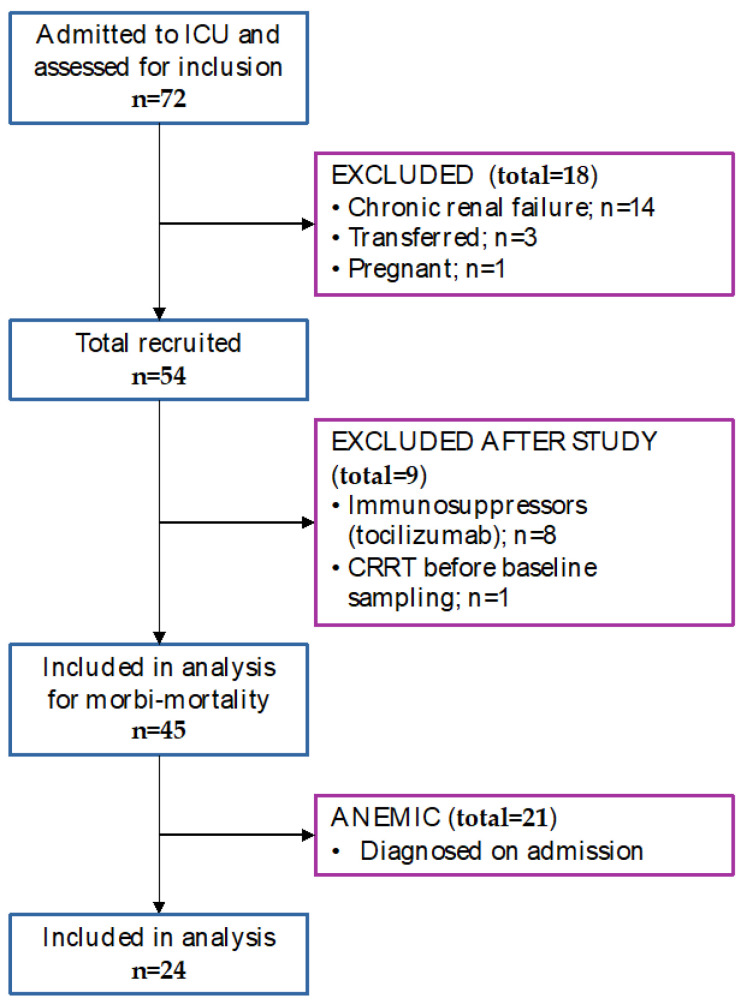
Flow diagram with numbers of patients assessed for eligibility, recruited, excluded, and analyzed.

During this period, 72 patients were admitted to ICU and were eligible for analysis. The study design is represented in Figure 1. A total of 18 patients were excluded from the study (14 presented chronic renal failure, three were transferred, and one was a pregnant woman). After inclusion further nine patients were excluded (eight had tocilizumab administered during ICU stay and one continuous renal replacement therapy initiated before baseline sampling).

### 3.2. Mortality

Of the 45 patients included in the analysis, 47% (*n* = 21) presented anemia on admission and were excluded based on laboratory findings. Of the non-anemic patients (*n* = 24), 13 (54%) survived and 11 (46%) died within 28 days of admission to ICU. Demographic, clinical, and laboratory variables on admission for the survivor (S) and non-survivor (NS) groups are presented in Table 1. Patients in NS compared to S group, were younger and presented higher comorbidity scores. Respiratory failure, infections or cardiac injury on admission were more prevalent in the NS group (*p* = 0.013, *p* = 0.047 and *p* = 0.104). Mean ferritin was significantly higher in NS compared to S (1802 mg/dL vs. 895.5 mg/dL; *p* = 0.039) while transferrin was lower (121.9 mg/dL vs. 163.4 mg/dL; *p* = 0.062). Lymphopenia and neutrophilia were more severe in NS with median lymphocyte count 0.57 × 10^9^/L; *p* = 0.027 and neutrophil count 14.66 × 10^9^/L; *p* = 0.074. Troponin levels were also higher in NS, 56.6 pg/mL compared to 8.7 pg/mL in S, *p* = 0.038. All other parameters at baseline were comparable between S and NS.

### 3.3. Multiple Organ Dysfunction

MOD was diagnosed on admission in 15 (63%) patients. The organ failure and laboratory values on admission are presented in Table 2. Respiratory and cardiac dysfunction were most prevalent (*p* = 0.003) followed by renal dysfunction (*p* = 0.01). Hematologic and liver dysfunction were not significantly more frequent between groups. In terms of laboratory parameters, hepcidin on admission was significantly higher in patients with MOD (328.7 pg/mL vs. 194.1 pg/mL; *p* = 0.034). Ferritin was also elevated in MOD patients however the significance was marginal (1507 ng/mL vs. 950.9 ng/mL; *p* = 0.11). Neither iron, transferrin saturation (TS), nor IL 6 were different between groups.

### 3.4. Anemia Following Admission

Anemia was the most prevalent comorbidity with 21 patients presenting low Hb on admission with further 17 developing anemia during ICU stay (total *n* = 38; 84%). Baseline parameters for patients who developed anemia during ICU (late anemia) and who did not develop anemia (no anemia) are shown in Table 3. When comparing patients who went on to develop late anemia (LA) and no anemia (NA), the former group presented with higher acute physiology and chronic health evaluation (17 vs. 8; *p* = 0.02) and Charlson comorbidity index (5 vs. 3; *p* = 0.057) scores. Hepcidin was increased in LA compared to NA (334.3 pg/mL vs. 162.1 pg/mL; *p* = 0.0275). Ferritin was not significantly higher in LA compared to NA (*p* = 0.7050). Mean Hb levels were also not different on admission (*p* = 0.7) however, the LA group presented higher TS (25.19%; *p* = 0.0316) and lower transferrin levels (126.5 mg/dL; *p* = 0.0488).

### 3.5. Anemia Requiring Transfusion

Further, we looked at patients who developed anemia and required blood transfusions. Hence, 29% of patients (*n* = 7) were transfused during ICU stay. In terms of serologic iron studies (Table 4), serum iron was significantly lower in transfused patients (25.17 ug/dL vs. 37.88 ug/dL; *p* = 0.0404) while ferritin and transferrin were borderline significant. Transfused patients had lower ferritin (638.0 ng/mL vs. 1264 ng/mL, *p* = 0.0604) and higher transferrin (174.0 mg/dL vs. 132.2 mg/dL, *p* = 0.0883) compared to non-transfused patients. The mean hepcidin level was 337.9 pg/mL in transfused patients, while in non-transfused, mean hepcidin was 219.8 pg/mL, (*p* = 0.0527).

### 3.6. Prediction of Outcome

**Figure 2:**
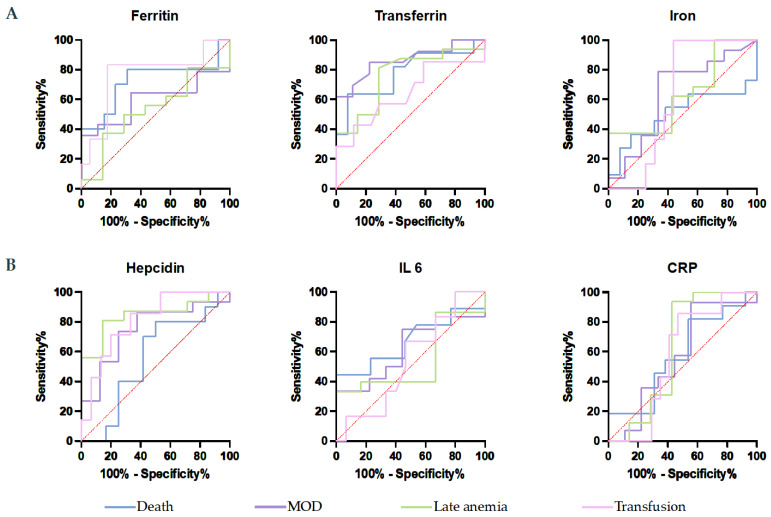
Predictive value of serologic iron (**A**) and inflammatory (**B**) parameters to identify non survivors, patients who developed MOD and LA. IL 6 = interleukin 6; CRP = C reactive protein; MOD = multiple organ dysfunction.

To evaluate the predictive value of inflammatory and iron parameters on admission, ROC curves were plotted (Figure 2). Ferritin cutoff level of 932.0 ng/mL was 80% sensitive and 69% specific for death with an AUC = 0.7231; *p* = 0.0721. Ferritin did not identify the occurrence of neither MOD nor LA (*p* = 0.59 and *p* = 0.78). Transferrin effectively identified non survivors, patients who developed MOD and LA. A value of 136.2 mg/dL presented a sensitivity of 84% and specificity of 77% for MOD, with an AUC = 0.8632 (*p* = 0.0045). Death was predicted with a sensitivity of 73% and specificity of 62%, AUC = 0.7797 (*p* = 0.02) and LA with 69% sensitivity and 71% specificity, AUC = 0.7634 (*p* = 0.0487). Iron did not demonstrate predictive values for either of the outcomes. Hepcidin was not efficient at predicting the occurrence of death. However, levels of 162.2 pg/mL identified LA patients with 87.5% sensitivity and 71.43% specificity, AUC = 0.8482 (*p* = 0.0092), and patients who developed MOD with a sensitivity of 87% and specificity of 63%, AUC = 0.75 (*p* = 0.0528). Neither IL 6 nor CRP predicted NS, MOD, or LA patients.

We also looked at the ability to predict transfusions using serologic parameters. Hepcidin of 256.4 pg/mL presented sensitivity of 85.71% and specificity of 66.67% with an AUC = 0.8095 (*p* = 0.022) while ferritin cut-off of 784.5 ng/mL presented an AUC = 0.7647 and sensitivity and specificity of 83.33% and 82.35% respectively (*p* = 0.0587). None of the other parameters predicted the need for transfusions.

### 3.7. Multi Variable Models to Predict Outcome

Finally, to better predict death, we assessed the combination of laboratory parameters (ferritin, transferrin, iron, and hepcidin) alone and with clinical variables. Block variables and the change from the beginning block (block 0) are shown in Table 5. Starting from a −2 Log Likelihood (−2*LL) of 52.09 for the model with no covariates (block 0), adding the laboratory parameters reduced the −2*LL to 45.27 (X^2^ = 6.82; *p* = 0.146). The addition of clinical parameters to laboratory parameters further improved the model to a −2*LL of 39.90 with cardiac injury (X^2^ = 11.96; *p* = 0.035), −2*LL of 45.05 with bacterial infection (X^2^ = 7.042; *p* = 0.218), −2*LL 45.27 with respiratory failure (X^2^ = 6.823; *p* = 0.234), −2*LL 44.36 with renal failure (X^2^ = 7.728; *p* = 0.171), and −2*LL 45.23 with MOD (X^2^ = 6.869; *p* = 0.231). Apart from the model which included laboratory variables and cardiac injury, no model was statistically significant.

## 4. Discussion

### 4.1. Oxidative Stress and Ferroptosis Are Associated with Multiple Organ Damage

COVID-19 activates the lung macrophages that release interleukins and ferritin and produce a self-perpetuating mechanism for the accumulation of ferritin, termed hyperferritinemia (HF) [13]. By producing organ damage, HF is associated with as much as a four-fold increase in fatality [14]. Inflammation mediator IL 6 plays a role in HF and elevated levels have been observed in critically ill patients, even from the moment of admission to ICU [15]. In our study ferritin and IL 6 levels were higher in non-survivors. The significance for IL 6 is marginal, possibly due to the small number of patients and the coexistence of bacterial infection in both groups. Finally, HF could also be produced by other mediators, such as IL 1, IL 18, and tumor necrosis factor (TNF) [15]. However, these mediators were not measured in our study.

HF leads to organ dysfunction but is not limited to the lungs. Iron mediated cell death, known as ferroptosis, produces multiple organ dysfunction where iron accumulates within cells and initiates a cascade of oxidative stress via Fenton reaction [16]. Lipid peroxidation of the cell membranes produces reactive oxygen species (ROS). The hydroxyl radicals, compounded by the depletion of antioxidants such as glutathione, represent the cause of the ensuing non-apoptotic cell death [6]. Pneumocyte ferroptosis also produces a shift in the coagulation-fibrinolysis balance favoring clot formation and stabilization in small capillaries [17]. Respiratory dysfunction seen in our patients may have occurred due to cell death and micro thrombosis secondary to ferroptosis [18]. Oxidative stress induced ferroptosis can also occur in other organs, such as the heart and kidneys, and is associated with increased mortality. In our study, respiratory failure and cardiac injury were most prevalent, followed by renal failure in patients with MOD. These findings are similar with other reports on COVID [19] and are supported by elevated troponin levels in our population of non-survivors.

### 4.2. Low Circulating Iron in Critical Illness Is Plurifactorial

Hypoferremia refers to low circulating iron and is detrimental even in the absence of anemia. Low serum iron develops when dietary intake is insufficient, when losses are present or when hepcidin is induced by inflammation [20]. Existing data suggest that low iron and TS are associated with COVID related mortality and morbidity. In our study, patients who did not survive or developed MOD, presented hypoferremia as early as admission. When compared with survivors and patients who did not develop MOD, no significant differences were observed. The absence of significance could be due to the confounding effects of gender. Besides lower serum iron seen in females, males tend to present with more severe forms of COVID and hence a more pronounced inflammatory status [21]. Another explanation could be that iron is lost through frequent blood sampling during hospital stay.

In our study, patients who developed anemia, despite low circulating iron, TS was normal while those who did not develop anemia presented low TS. This could be due to a very low transferrin compared to serum iron resulting in a pseudo normal TS. Transferrin was significantly lower in late anemia which in turn could be attributed to the inflammation present in this group. Cellular iron sequestration takes place with subsequent hypoferremia. Due to the lack of available iron for erythropoiesis, anemia develops and is termed inflammatory anemia [22]. When inflammatory and iron deficient anemia coexist, it is difficult to distinguish between the two. Elevated hepcidin, as seen in both late anemia and transfused groups, differentiates inflammatory from iron deficient anemia. Furthermore, the combination of high ferritin and hepcidin with low transferrin is associated with transfusions [23]. As an alternative to hepcidin, zinc protoporphyrin is another biomarker which identifies iron deficiency in mixt anemia and is not influenced by inflammation like ferritin and TS are [24]. Consequently, in these situations, clinicians are often faced with having to administer transfusions.

### 4.3. Hepcidin Is More Detrimental than Protective

Hepcidin levels were significantly higher in patients who developed MOD, anemia and required transfusions, but no difference was observed in terms of survival. Our results are in accordance with reports from a similar study, however the range of reported values was much lower in our study [14].

Factors which may have affected hepcidin levels are medication, age and disease related. All our patients received steroid therapy for anti-inflammatory effects and may have produced an overall down regulation of hepcidin. Moreover, enoxaparin was administered to all, adding to the list of hepcidin inhibitors. This could explain the lower levels reported by us. Different patients were treated with insulin, aspirin, vitamin D and spironolactone, all of which are inhibitors [18], possibly explaining why some results were marginal. Tocilizumab is a monoclonal antibody which inhibits IL 6 and reduces hepcidin [25]. IL 6 may also be decreased during continuous hemo-filtration. For these reasons, patients who received tocilizumab or underwent hemo-filtration were excluded from our analysis.

Hepcidin increases with age, possibly in association with the occurrence of hypertension, where angiotensin is incriminated. Our survivors were significantly older than the non-survivors and may have contributed to the lack of difference between groups. In terms of disease, obesity also contributes to higher levels, which we did not take into account when analyzing for differences. Of note, SARS-CoV-2 itself mimics hepcidin due to a similarity in amino acid chains producing hyperferritinemia and hypoferremia [21,26].

Hepcidin synthesis is signaled by IL 6, which makes the latter a molecule of interest in terms of targeted therapy. As an alternative to existent therapies, it would be of interest to see how targeting IL 6 and hepcidin or targeting overfilled iron stores using nanoparticles, could influence the outcome in COVID-19 [27]. Likewise, adopting a diet containing nutrients with antioxidant and anti-inflammatory properties, such as glutathion or selenium, could prove beneficial [28].

In contrast to our results, it was postulated that hepcidin, by sequestrating iron, plays a protective role in preventing iron from entering redox reactions. This could perhaps also explain why no difference was observed between survivors and non-survivors [26].

### 4.4. Strengths and Weaknesses

To our knowledge, this study is one of only a few prospective studies currently available where hepcidin and iron parameters have been assessed simultaneously in terms of mortality and disease severity. Likewise, it is the only study to look at anemia and transfusions in critically ill COVID patients. We are, however, aware of the inherent limitations. Being a pilot study, conducted on a small number of patients, limits out ability to draw any conclusions. The presence of many confounders in critical illness limits the significance of our results. Based on obtained difference in hepcidin values between no anemia and late anemia groups, we were able to calculate a minimum required sample size of 18 patients for each group (i.e., a total sample size of 36, assuming equal group sizes), to achieve a power of 80% and a level of significance of 5% (two sided). Therefore, the potential key markers and models identified here need further investigation in larger sample size studies to achieve a complete understanding of the pathophysiology behind COVID.

## 5. Conclusions

Some standard iron parameters, but not inflammatory markers, could be useful to determine mortality and morbidity. Ferritin on admission to ICU may predict mortality in COVID infected critically ill patients. Hepcidin, which is not routinely measured, may be used to predict multiple organ failure, anemia, and transfusions. Given the limitations of this study, neither individual parameters nor multi variable models can be regarded as accurate mortality predictors. Nonetheless, this paper brings to light new directions for further research.

## Figures and Tables

**Table 1:** Baseline characteristics of patients at enrollment.

	Survivor *n* = 13	Non-Survivor *n* = 11	
Demographic	*n* (%)		*p* value
Female	4 (31)	6 (55)	0.4081
Male	9 (69)	5 (45)	
	Mean (±SD)		
Age (years)	56 (±11.02)	47 (±10.98)	0.0435 *
Scores	Median (range)		
APACHE II	12 (7–18)	18 (15–21)	0.0645
CCI	3 (2–5)	4 (4–5)	0.1564
Clinical	*n* (%)		
Respiratory failure	5 (62)	10 (14)	0.0131 *
Infection	5 (58)	9 (43)	0.0472 *
Cardiac injury	6 (62)	9 (57)	0.1049
AKI	4 (31)	7 (64)	0.2173
Thrombocytopenia	1 (1)	3 (27)	0.3002
Liver dysfunction	0 (0)	2 (18)	0.1993
Pancreatitis	1 (1)	0 (0)	>0.9999
Thrombosis	1 (1)	1 (14)	>0.9999
Laboratory tests (unit)	Mean (±SD)		
Albumin (g/dL)	2.906 (±0.32)	2.552 (±0.66)	0.1313
CRP (mg/dL)	13.39 (±8.693)	17.96 (±13.24)	0.3407
Triglycerides (mg/dL)	215.8 (±79.83)	245.7 (±105.8)	0.4512
Ferritin (ng/mL)	895.5 (±497.1)	1802 (±1196)	0.0395*
Hepcidin (pg/mL)	255.0 (±166.5)	260.2 (±91.80)	0.6902
Transferrin (mg/dL)	163.4 (±52.07)	121.9 (±50.78)	0.0623
Fibrinogen (mg/dL)	516.2 (±220.8)	492.6 (±219.2)	0.7952
Leucocytes 10^9^/L	11.53 (±4.2)	15.93 (±8.35)	0.1343
Hemoglobin (g/dL)	13.73 (±0.98)	13.43 (±0.867)	0.4305
MCV (fL)	87.44 (±5.66)	89.29 (±4.09)	0.3639
Trombocytes (10^9^/L)	219.2 (±68.3)	221.8 (±103.5)	0.9443
Neutrophiles (10^9^/L)	9.804 (±4.295)	14.66 (±8.13)	0.0747
Limphocytes (10^9^/L)	1.14 (±0.72)	0.57 (±0.3)	0.0272 *
	Median (range)		
ALAT (IU/L)	30 (24.5–49)	32 (26–76)	0.5583
Direct bilirubin (mg/dL)	0.18 (0.14–0.25)	0.33 (0.13–0.48)	0.1537
Total bilirubin (mg/dL)	0.7 (0.44–0.88)	0.68 (0.52–1.05)	0.4843
LDH (IU/L)	404 (346.5–577)	535 (413–611)	0.1674
Iron (ug/dL)	29 (22.5–47)	27 (18–75)	0.9888
TS (%)	20 (13.50–26.25)	14.00 (9–49)	0.9156
Creatinine (mg/dL)	0.98 (0.89–1.22)	1.2 (0.79–2.49)	0.2665
Lipase (IU/L)	46 (29–66.5)	26 (13–44)	0.1299
Troponin I (pg/mL)	8.7 (5.9–30.5)	56.6 (18.55–105.7)	0.0381 *
Procalcitonin (<0.05)	0.14 (0.07–0.37)	0.34 (0.09–3.16)	0.3007
IL 6 (pg/mL)	16.10 (13.05–84.60)	83.00 (14.25–239.3)	0.1915

*n* = number; SD = standard deviation; APACHE II = Acute Physiology and Chronic Health Evaluation II; CCI = Charlson Comorbidity Index; AKI = acute kidney injury; ALAT = alanine aminotransferase; LDH = lactate dehydrogenase; TS = transferrin saturation; CRP = c-reactive protein; MCV = mean corpuscular volume; IL 6 = interleukin 6; * = statistically significant.

**Table 2:** Organ dysfunction and laboratory parameters on admission in patients without anemia.

	MOD		
	No (*n* = 9)	Yes (*n* = 15)	*p*-value
Organ dysfunction	*n* (%)		
Respiratory	2 (22)	13 (87)	0.003 *
Cardiac	2 (22)	13 (87)	0.003 *
Renal	1 (11)	10 (67)	0.0131 *
Hematologic	0 (0)	4 (27)	0.2589
Liver	0 (0)	2 (13)	0.5109
Laboratory parameters	Mean (±SD)		
Ferritin (ng/mL)	950.9 (±413.5)	1507 (±1157)	0.1181
Transferrin (mg/dL)	157.9 (±30.96)	114.5 (±30.63)	0.0039 *
CRP (mg/dL)	14.97 (±9.714)	13.56 (±8.675)	0.7196
Hepcidin (pg/mL)	194.1 (±89.22)	328.7 (±195.5)	0.0346 *
Hemoglobin (mg/dL)	13.39 (±0.6051)	13.71 (±1.074)	0.4176
	Median (range)		
Iron (ug/dL)	42.00 (25.75–58.69)	27.00 (20.84–56.22)	0.4548
TS (%)	21.50 (14.21–32.54)	14.50 (10.88–31.69)	0.3738
IL 6 (pg/mL)	15.60 (13.14–74.66)	45.35 (28.92–142.9)	0.3726

*n* = number; SD = standard deviation; TS = transferrin saturation; CRP = c-reactive protein; MCV = mean corpuscular volume; IL 6 = interleukin 6; * = statistically significant.

**Table 3:** Demographic, clinical and laboratory parameters of patients who developed anemia during ICU stay.

	General *n* = 24	No Anemia *n* = 7	Late Anemia *n* = 17	
Demographic	*n* (%)			*p* value
Female	10 (42)	4 (57)	6 (35)	0.3926
Male	14 (58)	3 (43)	11 (65)	
	Mean (±SD)			
Age (years)	52 (±11.83)	56 (±9.827)	50 (±12.37)	0.2499
Scores	Median (range)			
APACHE II	16 (8–20)	8 (5–16)	17 (13–23)	0.0204 *
CCI	4 (3–5)	3 (2–4)	5 (3–5)	0.0572
Laboratory tests (unit)	Mean (±SD)			
Hemoglobin (g/dL)	13.59 (±0.9250)	13.69 (±0.6517)	13.55 (±1.032)	0.7099
MCV (fL)	88.29 (±4.990)	86.37 (±3.079)	89.08 (±5.474)	0.1416
TS (%)	22.05 (±15.56)	13.67 (±6.439)	25.19 (±16.93)	0.0316 *
Ferritin (ng/mL)	1681 (±2137)	1171 (±871.2)	1342 (±1026)	0.7050
Hepcidin (pg/mL)	257.4 (±134.7)	162.1 (±84.05)	334.3 (±182.3)	0.0275 *
IL 6 (pg/mL)	67.91 (±74.31)	49.65 (±34.12)	75.22 (±85.29)	0.4904
CRP (mg/L)	14.12 (9.668-21.50)	17.70 (±13.13)	12.54 (±6.216)	0.2085
	Median (range)			
Iron (ug/dL)	29.00 (20.50–55.00)	26.00 (14.79–41.21)	29.00 (28.87–60.78)	0.2529
Transferrin (mg/dL)	132.5 (103.8–173.3)	160.5 (127.2–195.3)	126.5 (95.25–138.6)	0.0488 *
LDH (IU/L)	447.0 (358.8–583)	409.0 (365.1–546.3)	488.0 (392.2–723.5)	0.8042

*n* = number; SD = standard deviation; APACHE II = Acute Physiology and Chronic Health Evaluation II; CCI = Charlson Comorbidity Index; TS = transferrin saturation; CRP = c-reactive protein; MCV = mean corpuscular volume; IL 6 = interleukin 6; LDH = lactate dehydrogenase; * = statistically significant.

**Table 4:** Serologic parameters in not transfused (green) vs. transfused (purple) patients.

	Transfusion		
	No (*n* = 17)	Yes (*n* = 7)	*p*-value
Laboratory parameters	Mean (±SD)		
Transferrin (mg/dL)	132.2 (±37.86)	174.0 (±78.48)	0.0883
Iron (ug/dL)	37.88 (±22.00)	25.17 (±4.021)	0.0404 *
CRP (mg/dL)	16.25 (±12.80)	13.62 (±4.648)	0.4681
Hepcidin (pg/mL)	219.8 (±122.7)	337.9 (±131.3)	0.0527
	Median (range)		
Ferritin (ng/mL)	1264 (809–1750)	638.0 (307–1195)	0.0604
IL 6 (pg/mL)	50.70 (12.40–109.0)	26.30 (13.40–96.25)	0.8038

*n* = number; SD = standard deviation; TS = transferrin saturation; CRP = c-reactive protein; IL 6 = interleukin 6; * = statistically significant.

**Table 5:** Multivariate Cox proportional hazards analysis of different models and comparison with Block 0 or beginning block.

Model Variable	HR	95% CI	*p*-Value	−2*LL (X^2^)	*p*-Value
Block 1					
*Ferritin*	1.00	1.00–1.00	0.146		
*Iron*	0.98	0.97–1.00	0.207	45.27 (6.82)	0.146
*Hepcidin*	0.99	0.99–1.00	0.262		
*Transferrin*	0.99	0.98–1.00	0.296		
Block 2					
*Ferritin*	1.00	1.00–1.00	0.092		
*Iron*	0.98	0.96–1.00	0.091		
*Hepcidin*	0.99	0.99–0.99	0.363	39.90 (11.96)	0.035 *
*Transferrin*	0.98	0.99–0.98	0.424		
*Cardiac injury*	1.00	1.00–1.00	0.119		
Block 3					
*Ferritin*	1.00	1.00–1.00	0.134		
*Iron*	0.98	0.97–1.00	0.187		
*Hepcidin*	0.99	0.99–1.00	0.220	45.05 (7.042)	0.218
*Transferrin*	0.99	0.97–1.00	0.273		
*Bacterial Infection*	0.55	0.45–6.69	0.639		
Block 4					
*Ferritin*	1.00	1.00–1.00	0.163		
*Iron*	0.98	0.97–1.00	0.210	45.27 (6.823)	0.234
*Hepcidin*	0.99	0.99–1.00	0.299		
*Transferrin*	0.99	0.98–1.00	0.296		
*Respiratory failure*	1.02	0.09–11.59	0.988		
Block 5					
*Ferritin*	1.00	1.00–1.00	0.150		
*Iron*	0.98	0.97–1.00	0.188		
*Hepcidin*	0.99	0.99–1.00	0.262	44.36 (7.728)	0.171
*Transferrin*	0.99	0.98–1.00	0.346		
*Renal failure*	1.96	0.4238–11.17	0.404		
Block 6					
*Ferritin*	1.00	1.00–1.00	0.181		
*Iron*	0.99	0.97–1.00	0.238		
*Hepcidin*	0.99	0.99–1.00	0.291	45.23 (6.869)	0.231
*Transferrin*	0.99	0.98–1.00	0.303		
*MOD*	1.27	0.13–12.04	0.833		

HR = hazard ratio; CI = confidence interval; −2*LL = −2 Log Likelihood; X^2^ = Chi-square; MOD = multiple organ dysfunction; NOTE: Block 0 −2*LL is 52.09. * = statistically significant.

## Data Availability

Data is contained within the article.
